# Peer review of "Insect Meal Mixture as Sustainable Fishmeal Substitute in Rainbow Trout (Oncorhynchus mykiss) Diets"

_animals, 2025, doi:10.3390/ani15182661_

Round 1

Reviewer 1 Report

Comments and Suggestions for Authors

A brief summary:

This MS by Abdallah et al. investigates the effects of including a mixture of Hermetia illucens (HI) and Tenebrio molitor (TM) meals on feed color, growth performance, carcass yield, somatic indexes, nutrient digestibility, intestinal microbiota, histomorphometry and fillet quality of rainbow trout. The results suggest that partially defatted HI and full-fat TM meals, also in a mixture, can be considered suitable ingredients in low fish meal-based diets for rainbow trout. While pellet color reveals significant differences between treatments, dietary insect meal utilization had no adverse effects on growth performance, somatic indices and fillet chemical composition. No negative effects were observed on the overall physical quality of the fillets, although slight variations were noted in color lightness, pH, and water holding capacity.

  • General concept comments.

Firstly, I noticed that a high proportion of the cited references belong to authors: Refs.7,12,20,9,33,34,3,8,43,45,49,52,54, which is a self-citation rate of > about 21.67%. I think one of the reasons for the high self-citation rate may be there are too many authors listed (totally 14, and also no corresponding author marked here). I suggest that authors should retain important author information and make necessary deletions on authors number, or replace the self-cited references (Refs. 7,12,20,9,33,34,3,8,43,45,49,52,54).

Overall, I thought this was a good study in a system with limited previous knowledge of this level on this specific topic. I appreciated their approach and the time course involved in this work and think it add significant merit to their work. The Materials and Methods are basically clear and their overall conclusions were related to results. I think this work should undergo major revision before acceptance. I also have a couple suggestions.

  • Specific comments

Line 2  “rainbow trout” The fish name needs to be accompanied by its Latin name.

Line 92-100  Ref. 12 was self-cited reference, check it again.

Line 148-150  These sentences are the same as the annotations below Table 2 (142-145). They can be deleted or marked as "the same as above or idem or op. cit."

Line 164 What about the size of body length except for weight? Because the following will evaluate the condition factor (K) related to this index (Line 194).

Line 175 dissolved oxygen levels should be expressed the same as temperature, as means±SEM.

Line 191  “somatic indices (hepatosomatic index, (HSI)” Punctuation mark error. What is “somatic indices” ? Does it include HSI VSI CF?

Line 194 “(body length)3 (cm)” change to “body length (cm)3” The unit of K is g/cm3.

Line 349 In tab 4, it should be noted that the values are average values (means).

Line 350-353, 363-366, 376-379, 397-399, 443-445, 469-471, 477-479 ,483-485, 492-495These sentences are the same as the annotations below Table 2 (142-145). They can be deleted or marked as "the same as above or idem or op. cit."

Line 368, 380,400, 446, 473,480,486,497 “SEM, standard error of the mean” SEM has marked in Tab 4. There is no need to label and explain every time.

Line 455 The font size of the horizontal and vertical axes in the Fig 3 is too small to be legible. What does the “Emperer PCoA” mean in the Fig 3?

The Discussion is too long. The author could consider simplifying and retaining the important parts, or combine several parts to shorten the length.

Furthermore, the article content is too extensive. It is recommended to focus on discussing important factors with significant differences, and simplify those without significant differences.

Line 767-768 The author has already provided the abbreviations at the end of the article, so there is no need to explain them again in the text. This can also reduce the length of the article.

Author Response

1

General concept comments.

Firstly, I noticed that a high proportion of the cited references belong to authors: Refs.7,12,20,9,33,34,3,8,43,45,49,52,54, which is a self-citation rate of > about 21.67%. I think one of the reasons for the high self-citation rate may be there are too many authors listed (totally 14, and also no corresponding author marked here). I suggest that authors should retain important author information and make necessary deletions on authors number, or replace the self-cited references (Refs. 7,12,20,9,33,34,3,8,43,45,49,52,54).

We acknowledge the reviewer’s observation regarding the self-citation rate. We would like to clarify that our research group has been active in this field for many years and is considered one of the leading contributors. For this reason, some degree of self-citation is inevitable, as many of our previous works are directly relevant to the present study. Nevertheless, we have carefully revised the manuscript and reduced the number of self-citations by removing fewer essential references, while retaining only those that are fundamental for the context and discussion.

The following citations have been removed:

The following references have been added:

8- Bordignon et al 2020

34- Renna et al 2017

33- Elia et al 2018

43- Bruni et al 2018

45- Terova et al 2019

46- Antonopoulou et al 2019

17- Colombino et al 2023

8- Stadlander et al 2017

32- Cordineletti et al 2019

41- Drosdowech et al 2024

43- Cao et al 2024

46- Li et al 2021

59- Liland et al 2021

60- Chen et al 2022.

61- Eggink et al 2022

62- Tran et al 2022

Now the self-citation percentage should be equal to 12.90% [(8/62) x 100]

Overall, I thought this was a good study in a system with limited previous knowledge of this level on this specific topic. I appreciated their approach and the time course involved in this work and think it add significant merit to their work.

We sincerely thank the reviewer for their positive and encouraging feedback. We truly appreciate the recognition of our approach and the value of the time course included in this work, and we are pleased that the study is considered a meaningful contribution to this research area.

Specific comments

1- Line 2 “rainbow trout” The fish name needs to be accompanied by its Latin name. Corrected as requested.

2- Line 92-100 Ref. 12 was self-cited reference, check it again.

Checked as requested. We decided to keep it as it was considered relevant for the contextualization.

3- Line 148-150 These sentences are the same as the annotations below Table 2 (142-145). They can be deleted or marked as "the same as above or idem or op. cit."  

As the Tables and the manuscript text have to be read and understood separately from each other’s, all the relevant information has to be kept. Therefore, we respectfully decided to not change what we reported.

4- Line 164 What about the size of body length except for weight? Because the following will evaluate the condition factor (K) related to this index (Line 194).

Only the final length is needed for the condition factor (K), as K was calculated at the end of the experiment using the final body lengths of the fish. Therefore, the initial length of the fish was not measured.

5- Line 175 dissolved oxygen levels should be expressed the same as temperature, as means±SEM. Corrected as requested.

6- Line 191  “somatic indices (hepatosomatic index, (HSI)” Punctuation mark error. What is “somatic indices” ? Does it include HSI VSI CF? Corrected as requested.

7- Line 194 “(body length)3 (cm)” change to “body length (cm)3” The unit of K is g/cm3.

K is generally considered a dimensionless index (without unit). Reference: By R. Froese 2006. Cube law, condition factor and weight–length relationships: history, meta-analysis and recommendations. doi:10.1111/j.1439-0426.2006.00805.x.

8- Line 349 In tab 4, it should be noted that the values are average values (means).

All data are expressed as mean values. As this information is already reported in the Statistical Analysis subsection, it appears redundant to specify it again under Tables.

9- Line 350-353, 363-366, 376-379, 397-399, 443-445, 469-471, 477-479 ,483-485, 492-495These sentences are the same as the annotations below Table 2 (142-145). They can be deleted or marked as "the same as above or idem or op. cit."

As the Tables and the manuscript text have to be read and understood separately from each other’s, all the relevant information has to be kept. Therefore, we respectfully decided to not change what we reported.

10- Line 368, 380,400, 446, 473,480,486,497 “SEM, standard error of the mean” SEM has marked in Tab 4. There is no need to label and explain every time.

As the Tables and the manuscript text have to be read and understood separately from each other’s, all the relevant information has to be kept. Therefore, we respectfully decided to not change what we reported.

11- Line 455 The font size of the horizontal and vertical axes in the Fig 3 is too small to be legible. What does the “Emperer PCoA” mean in the Fig 3? Corrected as requested.  

The font size of the axes in Figure 3 has been increased for better readability. “Emperor PCoA” refers to the PCoA plot generated using the Emperor visualization tool, as clarified in the figure legend.

12- The Discussion is too long. The author could consider simplifying and retaining the important parts, or combine several parts to shorten the length. Furthermore, the article content is too extensive. It is recommended to focus on discussing important factors with significant differences, and simplify those without significant differences. Corrected as requested.  

Following the Reviewer’s suggestions, we shortened the Discussion in order to make it more concise and to-the-point.

14- Line 767-768: The author has already provided the abbreviations at the end of the article, so there is no need to explain them again in the text. This can also reduce the length of the article.

In the text, we provided the full name only at its first mention, and then used the abbreviation consistently throughout the article.

Reviewer 2 Report

Comments and Suggestions for Authors

The original manuscript provides insights into the experimental effects of adding insect-based additives to fish feed on the coloration of pellets, growth parameters, gut microbiota, and characterization of fillets in rainbow trout. In today's world, the use of specialized additives in fish feed can offer economic benefits for fish farms due to their low cost and specific bioactive properties that may enhance fish production and health. In the present study, the authors concluded that after 84 days of dietary treatment with different diets, some changes in feed pellet and fillet color were observed, while the overall performance and quality parameters of the cultivated fish remained unaffected. This suggests the potential to replace expensive fishmeal with more affordable insect meal while maintaining high-quality fish production. The study employs a comprehensive set of methodological diagnostics that clearly support the findings. Thus, I believe that after minor revisions, the manuscript could be published in Animals.

Nevertheless, I recommend that the authors carefully improve the manuscript and data before publication. Additionally, I noticed discrepancies in the percentages of additives used in each treatment, and I am impressed by the authors' thorough explanation of these in the manuscript. Please see my comments below.

Abstract

Please consider how this section could be simplified. I believe many methodological details could be moved to the M&M section.

Line 34. Please add the Latin name of rainbow trout.

Line 22. Abbreviations are unnecessary here; please delete them.

Line 38. Please place the abbreviation for grams (g) after the value 1.71. I believe it is more important to mark that you studied juvenile fish with an average weight of 126 g.

Lines 43–46. I think that the pellet color was influenced by the additives (HI and TM), rather than the diet itself; please clarify. Additionally, it would be helpful to specify what color changes were observed. Please explain what the parameter pH24h refers to.

Introduction

Dear authors, you provide a good review of the positive effects of diets with HI and TM additives on rainbow trout. Additionally, please clarify the rationale for conducting this study, given that all results showed these positive effects. Did you hypothesize that a more in-depth investigation was necessary, or did you aim to assess the specific response of fish to diets with insect additives? What is the main justification for your study?

Line 90. I believe you mean the liver cytological status or a similar parameter, as histology is a method rather than an outcome.

Line 104. Please clarify here why pellet color is an important parameter. You only provided this explanation in the Discussion (Lines 500–503).

M&M

Lines 123–217: Please simplify this sentence. While I understand that you replaced all or part of the fishmeal (FM) in the experiments, the current sentence is difficult to read. Based on Table 2 (Columns 3 and 4), more than 100% of FM (15 g per kg) was replaced by HI (18.6 g per kg) and TM (21.85 g per kg). The other percentages in the Table also do not correspond accordingly. Please clarify how you calculated the 25–100% of additives. Additionally, please provide the producer information for the fishmeal.

Table 3 should be moved to the Supplementary materials, as it is not explained in the main text.

Lines 173–175: Please correct this sentence for clarity.

Results

Line 341: Please delete the first sentence and include the citation for Table 4 in the following sentence. Did you use the average SEM for all groups? I am doubtful about the necessity of including thus type of SEM in the tables here and elsewhere. It would be better to report individual SEM values for each experimental group.

Line 362: Please clarify what "n" indicates here and elsewhere. Does it refer to the number of replicates or the number of fish analyzed in each trial?

Line 371: Please ensure that the paragraph does not start with a citation to the tables and figures. Additionally, it seems that the citation for Figure 3 was not included in the text; please check and correct this.

Author Response

Review 2

Comments and Suggestions for Authors

Abstract

1- Please consider how this section could be simplified. I believe many methodological details could be moved to the M&M section. Corrected as requested.  

2- Line 34. Please add the Latin name of rainbow trout. Corrected as requested.  

3- Line 22. Abbreviations are unnecessary here; please delete them. Corrected as requested.  

4- Line 38. Please place the abbreviation for grams (g) after the value 1.71. I believe it is more important to mark that you studied juvenile fish with an average weight of 126 g. Corrected as requested.

5- Lines 43–46. I think that the pellet color was influenced by the additives (HI and TM), rather than the diet itself; please clarify. Additionally, it would be helpful to specify what color changes were observed. Corrected as requested.

6- Please explain what the parameter pH24h refers to. Corrected as requested.

Introduction

7- Dear authors, you provide a good review of the positive effects of diets with HI and TM additives on rainbow trout. Additionally, please clarify the rationale for conducting this study, given that all results showed these positive effects. Did you hypothesize that a more in-depth investigation was necessary, or did you aim to assess the specific response of fish to diets with insect additives? What is the main justification for your study? Implemented as requested.

8- Line 90. I believe you mean the liver cytological status or a similar parameter, as histology is a method rather than an outcome. Corrected as requested.

9- Line 104. Please clarify here why pellet color is an important parameter. You only provided this explanation in the Discussion (Lines 500–503). Corrected as requested.

M&M

10- Lines 123–217: Please simplify this sentence. Corrected as requested.

The six experimental diets were designed with different inclusion levels HI, TM and the combination (HI and TM) while maintaining the same crude protein, ether extract and gross energy contents.

While I understand that you replaced all or part of the fishmeal (FM) in the experiments, the current sentence is difficult to read. Based on Table 2 (Columns 3 and 4), more than 100% of FM (15 g per kg) was replaced by HI (18.6 g per kg) and TM (21.85 g per kg). The other percentages in the Table also do not correspond accordingly. Please clarify how you calculated the 25–100% of additives. Additionally, please provide the producer information for the fishmeal.

We thank the reviewer for this important comment and the opportunity to clarify. In our study, fishmeal was completely replaced by HI and TM meals in diets HI100 and TM100. However, since the protein content of HI and TM meals was lower when compared to FM, higher inclusion levels were necessary in order to maintain isonitrogenous diets. Due to the different chemical composition of HI and TM relative to FM, and to ensure that the diets remained isonitrogenous, isolipidic, and isoenergetic, in the HI100 and TM100 diets with total FM replacement, the inclusion of wheat starch was slightly reduced. As indicated in line 122, the experimental diets were commissioned, formulated, and extruded by Sparos LDA (Olhão, Portugal). Therefore, we do not have the detailed description of the fishmeal used. We have requested this information from the producer but, at the moment, we have not yet received a reply.

11- Table 3 should be moved to the Supplementary materials, as it is not explained in the main text. Corrected as requested.

The table 3 has been moved to a separate document and is now referred to as Table S1 in the Supplementary Material S1.

11- Lines 173–175: Please correct this sentence for clarity. Throughout the experiment, rainbow trout were reared under controlled conditions with a water flow rate of 8 L/min per tank, a temperature of 14 ± 1 °C, and dissolved oxygen levels ranging from 8.6 to 9.5 mg/L. Corrected as requested.

Results

12- Line 341: Please delete the first sentence and include the citation for Table 4 in the following sentence. Corrected as requested.

13- Did you use the average SEM for all groups? I am doubtful about the necessity of including thus type of SEM in the tables here and elsewhere. It would be better to report individual SEM values for each experimental group.

The utilization of the pooled SEM is a common finding in animal nutritional studies. Therefore, we respectfully asked the Reviewer to keep this dispersion measure to be consistent with the available scientific literature.

14- Line 362: Please clarify what "n" indicates here and elsewhere. Does it refer to the number of replicates or the number of fish analyzed in each trial? Corrected as requested.

15- Line 371: Please ensure that the paragraph does not start with a citation to the tables and figures. Corrected as requested.

16- Additionally, it seems that the citation for Figure 3 was not included in the text; please check and correct this. Corrected as requested.

Reviewer 3 Report

Comments and Suggestions for Authors

  • The authors conducted a feeding study and extensive analyses to investigate the potential of the two insect meals.
  • They concluded that these insect diets had no adverse effects on fish growth, digestibility, gut microbiota, or liver and intestinal histopathology, but some of their methods and conclusions are questionable.

Major points

  • The growth parameters of the control group in this study seem to be inferior to those in reports by other researchers. For example,
    • 34) Renne et al. 2007. Initial wt.178g, duration 78days, SGR1.4, WGR 201%
    • 12) Biasato et al. 2022. 113g, 133days, SGR 0.9, 315%
    • Present study, Abdallah et al. 2025. 129g, 99days, SGR 0.8, 97%
    • (WGR (% weight gain rate)= WG/IBW x100)
  • The lower growth performance in the present study may be due to problems with the fish, the rearing environment, or the feeds.
  • Isn't the reason there were no differences in growth between the control and test groups that the fish in the control fish didn't grow appropriately? Wouldn't it be invalid to use such a poorly performing control and compare it to the test group?

Table1.

  • The EE and CP of HI are 26.16 and 52.06, while those of TM are 7.94 and 46.90, yet GE of HI is 20.84, which is lower than TM 25.44. Why is the energy value of lower nutrient content higher than that of higher one?
    • It doesn't make sense. Moreover, the EE seems quite high for the partially extracted HI. Is this value correct?

Table 3

  • Based on the values ​​in Tables 1 and 2, the EE content of the HI100 diet is approximately 22.8.: HI meal 4.9 (18.6 * 0.2616) + fish oil 7.00 + rapeseed oil 10.90 = 22.8
  • The EE content of TM100 diet is calculated approximately 17.2.: TM meal 1.7 (21.85 * 0.0794) + fish oil 7 + rapeseed oil 8.50 = 17.2.  In Table 3, the value for HI100 is 22.15, which is close to the calculated value above. However, the value of Table 3 for TM100 is 22.24, which is significantly lower than the calculated value of 17.2. Could you explain why this happened?
  • The authors focus on pellet color, but I don't understand why this test is important. It is difficult to reproduce the current results because pellet color depends on the raw material, its combination with other ingredients, its content, and the pellet processing temperature. Therefore, it is difficult to generalize the current results and apply them to other studies.
  • Additionally, actual data such as feeding rate (g/fish/min) and feed amount (g/fish/wt) are desirable

Microbiota

  • What is the implication of the microbiota data?
  • For example, is diverse flora an indication of a better condition of the intestine (fish health)?
  • Or, since the present results showed no difference between the control and insect group the different microflora not show fish health? It would be beneficial for the reader to not only present the results but also the author's comments on those results.

Minor points

  • L410 “Additionally, Figure 3 presents the Principal Coordinates …..”  Figure numbers should be written in the order in which they appear in the text. “Distribution of bacteria with Beta analysis…”, originally denoted as Figure 3 should be changed to Figure 1.
  • According to the authors, the study aimed to investigate the synergistic effects of two insect meals on rainbow trout (L102). The authors should mention the synergistic results. So, the authors should mention potential synergies in their conclusions.
  • L720-724 “Renna et al. (34) reported…. This decline in digestibility …was attributed to the increased chitin content….” This inference may need to be reconsidered. In Renna’s study, the value of H50 was not statistically different from HI0 (no chitin). This indicates that chitin may not be attributed to the reason for lower digestibility.

Author Response

Reviewer 3

The authors conducted a feeding study and extensive analyses to investigate the potential of the two insect meals. They concluded that these insect diets had no adverse effects on fish growth, digestibility, gut microbiota, or liver and intestinal histopathology, but some of their methods and conclusions are questionable.

Major points

1- The growth parameters of the control group in this study seem to be inferior to those in reports by other researchers. For example,

34) Renne et al. 2007. Initial wt.178g, duration 78days, SGR1.4, WGR 201%

12) Biasato et al. 2022. 113g, 133days, SGR 0.9, 315%

Present study, Abdallah et al. 2025. 129g, 99days, SGR 0.8, 97%

(WGR (% weight gain rate) = WG/IBW x100)

The lower growth performance in the present study may be due to problems with the fish, the rearing environment, or the feeds. Isn't the reason there were no differences in growth between the control and test groups that the fish in the control fish didn't grow appropriately? Wouldn't it be invalid to use such a poorly performing control and compare it to the test group?

In our study, all fish were reared under identical conditions, including stocking density, temperature, dissolved oxygen, photoperiod, and in the same rearing facilities, and were fed the seven tested diets. The differences in growth observed compared to other studies may reflect variations related to the trout strain used (different fish sourcing farms), the season during which the experiment was conducted, as well as the water source used in our experiment.

2- Table 1: The EE and CP of HI are 26.16 and 52.06, while those of TM are 7.94 and 46.90, yet GE of HI is 20.84, which is lower than TM 25.44. Why is the energy value of lower nutrient content higher than that of higher one? It doesn't make sense. Moreover, the EE seems quite high for the partially extracted HI. Is this value correct?

 We appreciate the reviewer for highlighting this issue. Upon careful verification, we identified a typographical error in the manuscript: the reported values for ether extract (EE) and crude protein (CP) were inverted between TM and HI meals. The correct proximate composition is as follows: TM meal (full-fat) – EE 26.16%, CP 52.06%; HI meal (partially defatted) – EE 7.94%, CP 46.90%. These corrected values have been updated in the manuscript.

3- Table 3 (table S1 Supplementary material): Based on the values ​​in Tables 1 and 2, the EE content of the HI100 diet is approximately 22.8.: HI meal 4.9 (18.6 * 0.2616) + fish oil 7.00 + rapeseed oil 10.90 = 22.8.

The EE content of TM100 diet is calculated approximately 17.2.: TM meal 1.7 (21.85 * 0.0794) + fish oil 7 + rapeseed oil 8.50 = 17.2.  In Table 3, the value for HI100 is 22.15, which is close to the calculated value above. However, the value of Table 3 for TM100 is 22.24, which is significantly lower than the calculated value of 17.2. Could you explain why this happened?

Thank you for your valuable comment. You are correct in noting the inconsistency. Since the feed analyses were performed in our laboratories, we are confident in the accuracy of the reported values. The diets were specifically formulated to be isoenergetic, isoproteic, and isolipidic. The most plausible explanation is that the batches of HI and TM meals supplied to us by the two companies (and analyzed in our lab, with the values originally reported in Table 1) may have differed from those sent directly to Sparos for diet preparation. As is well known, the production process of insect-based meals is not yet fully standardized, and substantial compositional differences may occur between different batches.

Given that the manuscript focuses on the experimental results obtained with the seven diets rather than on the proximate composition of the insect meals themselves, we have decided to remove Table 1.

3- The authors focus on pellet color, but I don't understand why this test is important. It is difficult to reproduce the current results because pellet color depends on the raw material, its combination with other ingredients, its content, and the pellet processing temperature. Therefore, it is difficult to generalize the current results and apply them to other studies.

We appreciate the Reviewer’s comment. The evaluation of pellet color was included because feed appearance can influence feed acceptance and feeding behavior in fish, as previously reported in aquaculture studies. We agree that pellet color may be influenced by several factors such as the type of raw materials, ingredient composition, nutrient content, and processing conditions. For this reason, our results should not be generalized beyond the present experimental context.

Additionally, actual data such as feeding rate (g/fish/min) and feed amount (g/fish/wt) are desirable.

We thank the reviewer for the suggestion. However, we believe that reporting the feeding rate alone (1.32% of body weight) is sufficient to describe the feeding conditions in this experiment, as it accurately reflects both the amount of feed provided and the feeding management.

Microbiota

4- What is the implication of the microbiota data? For example, is diverse flora an indication of a better condition of the intestine (fish health)? Or, since the present results showed no difference between the control and insect group the different microflora not show fish health? It would be beneficial for the reader to not only present the results but also the author's comments on those results.

We thank the reviewer for this suggestion. We have added the following sentence in the microbiota section of the Discussion to interpret the results: In this study, the gut microbiota showed a positive shift toward SCFA-producing bacteria and reduced foodborne pathogens, suggesting a beneficial increase in diversity associated with improved ability of fish to cope with intestinal disorders [12].”

Minor points

5- L410 “Additionally, Figure 3 presents the Principal Coordinates …..”  Figure numbers should be written in the order in which they appear in the text. “Distribution of bacteria with Beta analysis…”, originally denoted as Figure 3 should be changed to Figure 1. Corrected as requested.

6- According to the authors, the study aimed to investigate the synergistic effects of two insect meals on rainbow trout (L102). The authors should mention the synergistic results. So, the authors should mention potential synergies in their conclusions. Corrected as requested.

7- L720-724 “Renna et al. (34) reported…. This decline in digestibility …was attributed to the increased chitin content….” This inference may need to be reconsidered. In Renna’s study, the value of H50 was not statistically different from HI0 (no chitin). This indicates that chitin may not be attributed to the reason for lower digestibility. Corrected as requested.

We agree with the Reviewer. Therefore, we removed this Reference.

Round 2

Reviewer 1 Report

Comments and Suggestions for Authors

Line 184  “HIS” change to "HSI"

The author has made point-by-point revisions according to the reviewer's requirements. I agree to revise and publish  in accordance with the journal's format requirements.

Reviewer 3 Report

Comments and Suggestions for Authors

No further comments.