# Supplementary material for: Insect Meal Mixture as Sustainable Fishmeal Substitute in Rainbow Trout (Oncorhynchus mykiss) Diets
Source: Animals (Basel). 2025 Sep 11;15(18):2661. doi: 10.3390/ani15182661 (PMC12466574; doi:10.3390/ani15182661)
Supplement: Supplementary file 1 [file animals-15-02661-s001.zip › animals-3845315-supplementary.pdf]

Supplementary Materials

Table S1. Chemical composition, essential amino acids (EAAs), non-essential amino acids (AAs), and other components of the seven experimental diets.

Items	Experimental diets						
	**CTRL**	**HI100**	**TM100**	**MIX25**	**MIX50**	**MIX75**	**MIX100**
Proximate composition, g/100g as fed unless stated							
DM	96.52	97.00	96.18	96.41	96.25	96.28	96.09
Ash	7.05	7.19	6.94	7.00	6.91	7.01	6.92
CP	50.38	50.89	51.15	50.27	50.00	51.17	50.31
EE	22.07	22.15	22.24	22.73	22.48	22.31	22.27
Chitin	-	1.64	2.00	0.94	1.21	1.65	1.89
GE, MJ/kg as fed	23.15	23.51	23.51	23.61	23.48	23.64	23.91
EAAs, g/100g as fed							
Arginine	2.5	2.5	2.5	2.5	2.4	2.4	2.4
Histidine	1.0	1.0	1.0	1.0	1.0	1.0	1.0
Isoleucine	1.7	1.7	1.8	1.7	1.7	1.7	1.7
Leucine	3.5	3.3	3.5	3.4	3.4	3.4	3.4
Lysine	2.5	2.5	2.5	2.5	2.5	2.5	2.5
Threonine	1.6	1.6	1.7	1.6	1.6	1.6	1.6
Tryptophan	0.5	0.5	0.5	0.5	0.5	0.5	0.5
Valine	2.0	2.1	2.2	2.0	2.1	2.1	2.1
Methionine	0.8	0.8	0.8	0.8	0.8	0.8	0.8
Cysteine	0.6	0.6	0.6	0.6	0.6	0.6	0.6
Methionine + Cysteine	1.5	1.4	1.4	1.4	1.4	1.4	1.4
Phenylalanine	2.1	2.0	2.1	2.1	2.1	2.1	2.1
Tyrosine	1.4	1.7	2.0	1.5	1.7	1.8	1.9
Phenylalanine + Tyrosine	3.5	3.7	4.1	3.6	3.7	3.8	3.9
AAs, g/100g as fed							
Aspartic acid	3.6	3.5	3.7	3.6	3.6	3.6	3.6
Glutamic acid	9.1	8.8	9.1	9.1	9.0	9.0	9.0
Alanine	2.2	2.2	2.4	2.2	2.2	2.3	2.3
Glycine	2.4	2.1	2.2	2.3	2.3	2.2	2.2
Proline	3.0	3.1	3.3	3.0	3.1	3.1	3.2
Serine	2.1	2.1	2.1	2.1	2.1	2.1	2.1
Other compositions, g/100g as fed							
Taurine	0.1	0.0	0.0	0.1	0.1	0.1	0.0
Total phosphorus	1.0	1.0	1.0	1.0	1.0	1.0	1.0
EPA + DHA	2.0	2.0	2.0	2.0	2.0	2.0	2.0

Abbreviations: CTRL, control diet; HI100, 100% of fish meal replaced by HI meal; TM100, 100% of fish meal replaced by TM meal; MIX25, MIX50, MIX75, and MIX100, respectively, with 25%, 50%, 75%, and 100% of fish meal replaced by a mixture (1:1) of HI and TM meals; DM, dry matter; CP, crude protein; EE, ether extract; GE, gross energy; EPA, eicosapentaenoic acid; DHA, docosahexaenoic acid; AA, amino acid; (-), not present.
